# Analysis of the efficiency and sustainability of the underground loop in Hangzhou Future Science and Technology City

**DOI:** 10.1371/journal.pone.0352815

**Published:** 2026-07-14

**Authors:** Huajin Zhang, Qifeng Yu

**Affiliations:** 1 Shanghai Municipal Engineering Design Institute (Group) Co., Ltd., Shanghai, China; 2 College of Transport & Communications, Shanghai Maritime University, Shanghai, China; Wuhan University of Technology, CHINA

## Abstract

In order to improve the low utilization efficiency and limited environmental benefits of the underground loop in Hangzhou Future Science and Technology City, this study systematically analyzes the operation status of the loop road by collecting traffic flow data through field research and combining questionnaire interviews to understand the driver’s experience. It is found that the current underground loop system faces several challenges, including low public awareness, insufficient precision in traffic guidance, inconsistent management of land parcel access interfaces, limited operational flexibility, and inadequate realized carbon reduction. As a result, only about 10% of traffic uses the loop for arrival and departure, and the utilization of underground transportation resources remains far below the expected target. Based on the comparative analysis of similar projects such as Xidong New City in Wuxi and Zhenru Subcenter in Shanghai, this study proposes strategies such as constructing a comprehensive four-tier traffic guidance system, strengthening intelligent information management and control, unifying the collaborative management standards of parcel access interfaces, and deepening low-carbon and sustainable operation. These strategies can effectively alleviate surface-level traffic, provide practical references for the planning and operation of urban underground transportation facilities, and help the core area realize the synergistic and sustainable development of transportation and space.

## Introduction

Hangzhou Future Science and Technology City, located in Yuhang District of Hangzhou, constitutes an important part of the Zhejiang Overseas High-Level Talents Innovation Park and serves as a core area in the development of the Chengxi Sci-Tech Innovation Corridor. As a key carrier for the digital economy and sci-tech innovation, it accommodates a diversified range of industrial functions, including headquarters economy, scientific research and development, and entrepreneurship and innovation. On 10 May 2024, Alibaba’s Global Headquarters was officially inaugurated here, and the Cainiao Network Headquarters, along with other key enterprises, subsequently settled in. These developments have not only strengthened the area’s advantages in attracting high-end industries and talents but have also generated frequent and high-intensity travel demand, resulting in strong external connectivity and an active pattern of business activities.

Under conditions of high industrial agglomeration, the traffic demand of Hangzhou Future Science and Technology City exhibits distinct structural characteristics. First, a pronounced tidal commuting pattern is observed, with a large inflow of vehicles during the morning peak and a concentrated outflow during the evening peak, reflecting strong directional flow. Second, the demand for high-frequency business travel is significant, accompanied by high requirements for timeliness and accessibility. Third, during holidays and large-scale events, commercial complexes and cultural and leisure facilities substantially increase regional travel volumes. These characteristics collectively exacerbate traffic pressure during peak periods and impose higher requirements on the efficiency, capacity, and flexible scheduling of the traffic system.

At present, the surface-level traffic system in the core area faces several structural constraints. First, roadway cross-sections are generally narrow, limiting traffic capacity. Second, through traffic and arrival-and-departure traffic interfere with one another both spatially and temporally, affecting the efficiency of internal traffic organization. Third, parking facilities are scattered, and the management of parcel access interfaces lack unified standards, leading to uneven resource utilization. These issues jointly undermine the overall operational performance of the traffic system, and without systematic intervention, such conflicts are expected to intensify with the continued expansion of industrial scale and population, thereby constraining the vitality of regional industrial development.

In this context, the construction and improvement of the underground loop is not merely an engineering measure to alleviate surface-level traffic pressure, but also an essential infrastructure for ensuring the efficient operation of the core area. By vertically separating traffic flows, arrival-and-departure traffic can be directed underground for distribution, thus reducing surface traffic conflicts. In addition, direct connections to underground garages of parcels facilitate the optimized utilization of parking resources and reduce the cost of surface detours. Furthermore, freeing up surface space and improving the public environment provide important support for achieving low-carbon development goals in the region. Therefore, enhancing the operational efficiency of the underground loop is not only integral to refining the regional traffic system, but is also closely linked to strengthening competitiveness and promoting the sustainable development of Hangzhou Future Science and Technology City in the digital economy era.

Building on this context, the present study analyzes the operational status of the underground loop in Hangzhou Future Science and Technology City, identifies key challenges, and proposes targeted strategies to enhance its utilization efficiency and support the sustainable development of the core area.

## Literature review

The concept of underground loops can be traced back to the 1930s, when their original purpose was to preserve the natural spatial structure of cities and alleviate surface traffic congestion [1]. With the continuous growth of motor vehicle numbers, underground loops have gradually become an essential component of urban transportation systems [2]. International practices demonstrate varied outcomes: the Shinjuku underground loop in Tokyo has achieved remarkable success in the efficient integration of surface and underground traffic, though improvements in intelligent guidance remain necessary; Singapore’s Marina Bay project emphasizes smart management but falls short in multi-plot coordination mechanisms; and the Bagubai underground loop has effectively reduced through traffic, though it lacks sufficient attention to green sustainability indicators [3]. A review of existing studies shows that scholars have systematically examined facility evolution and policy challenges, laying a theoretical foundation [4]. However, a comprehensive solution that integrates traffic operations, spatial coordination, and sustainable management has yet to be developed.

(1) Traffic Guidance

Traffic guidance systems are widely regarded as a core means of improving the operational efficiency of underground loops. Driving simulation experiments have shown that appropriate advance warnings and visual guidance can significantly enhance exit-finding efficiency and driving stability while avoiding guidance failure due to information overload [5]. In diverging and merging areas, integrated guidance schemes have been validated to improve traffic safety and flow, supported by simulation-based driving experiments in urban underground road scenarios [6]. Road tests under real-world conditions have revealed correlations between driving risks on curved underground sections and geometric parameters, underscoring the role of visual guidance in risk mitigation [7]. In mixed traffic scenarios simulating enclosed underground environments, the effects of different coordination strategies on efficiency and safety have been evaluated, with new measures such as path overlap density proposed for underground traffic systems [8].

Existing studies mainly emphasize single schemes or localized testing, with limited attention to multi-level guidance layouts and the coordination between underground and surface traffic. Most rely on simulations or small-scale pilots, lacking comprehensive evaluation of full-loop operations. In view of this gap, this study focuses on designing and assessing a four-tier traffic guidance system that integrates surface–underground information coordination, aiming to improve loop utilization and operational efficiency in practice.

(2) Parcel Coordination

In recent years, increasing academic attention has been paid to the coordinated development of underground loops and adjacent land parcels. Under the data-driven paradigm of urban underground space planning, early-stage planning should ensure the deep integration of surface land use with underground traffic functions, supported by cross-stakeholder governance mechanisms to achieve coordinated multi-parcel layouts [9]. For underground spaces around metro stations, an *NSGA-II*-based intelligent layout model has been developed to balance land-use conflicts, development benefits, and construction scale, thereby facilitating spatial coordination among multifunctional and multi-ownership plots [10]. In urban complex contexts, shared parking utility models have validated the potential for time-based parking space sharing across different land uses, offering empirical evidence for cross-plot parking garage interconnection and joint scheduling [11]. Multi-objective optimization approaches have also been applied to the siting of smart underground parking facilities in high-density urban areas, demonstrating the importance of integrated resource allocation from the dual perspectives of accessibility and system efficiency [12]. From an institutional perspective, governance studies have highlighted that standardized interfaces and operational coordination are critical for successful cross-parcel collaboration [13].

Existing studies highlight the need for integrating underground loops with adjacent parcels, but most focus on single plot types or isolated cases. Comprehensive frameworks for cross-parcel coordination, standardized interfaces, and joint scheduling remain underdeveloped. This study therefore investigates parcel-loop interconnection in Hangzhou, emphasizing unified interface standards and collaborative management mechanisms to enhance efficiency and coordination across multiple parcels.

(3) Low-Carbon Operations and Sustainability

With regard to energy conservation and emission reduction, existing studies have highlighted the potential of underground loops and broader underground space infrastructure to reduce carbon emissions from multiple perspectives. Rational development of urban underground space is considered an effective means to cut carbon emissions associated with road operations and to promote the transformation of transportation systems toward sustainability [14]. For complex underground road operations, Ma and Peng [15] systematically summarized sustainability benefits, covering traffic efficiency, energy use, and environmental improvements. In quantitative carbon emission assessments, comparative studies between a new 1000 m tunnel route and an existing mountain pass route, combined with Life-Cycle Assessment (LCA) methods, have confirmed that underground infrastructure can reduce fuel consumption and CO₂ emissions during operations by minimizing detours and steep gradients [16]. Air quality monitoring further reveals that following the opening of the Eurasia Undersea Highway Tunnel, average concentrations of pollutants such as CO, PM₁₀, and PM₂.₅ at nearby surface monitoring sites decreased by 12 ~ 46%, indirectly reflecting reduced surface congestion and lower pollutant exposure levels after traffic was diverted underground [17].

Beyond road tunnels, recent studies on underground space have further enriched the understanding of low-carbon operations from complementary angles. Research on policy incentives demonstrates that well-designed carbon pricing and subsidy mechanisms can effectively shift transport demand toward underground infrastructure, yielding both cost savings and emission reductions [18]. Other work has highlighted the indirect ecological benefit of underground space use: by freeing up surface land for blue-green spaces, underground development can create additional urban carbon sinks [19]. From a system perspective, analyses of coordination between transport infrastructure and regional socio-economic development suggest that enhancing such coupling can improve long-term sustainability [20], while proposals for multi-energy integration in underground transit systems point to pathways for increasing low-carbon resilience [21]. Furthermore, improvements to the built environment around underground transport nodes have been shown to encourage public transit use and reduce private vehicle emissions [22,23]. Taken together, these findings move beyond single-segment tunnel assessments and underscore the importance of policy design, land-use synergy, system coordination, and traveler behavior in realizing the carbon-reduction potential of underground loops.

Overall, prior studies on underground loops have provided valuable insights into traffic guidance, parcel coordination, and sustainability, yet they remain fragmented and localized. Research on traffic guidance often stops at single-scheme or pilot experiments, parcel coordination lacks unified standards and cross-parcel mechanisms, and low-carbon assessments seldom integrate operational strategies across the whole loop. Building on these gaps, this study aims to answer three key questions: (1) what are the current utilization patterns and operational challenges of the underground loop in Hangzhou Future Science and Technology City; (2) how can traffic guidance, parcel coordination, and low-carbon strategies be optimized to improve efficiency; and (3) what lessons can be drawn from comparable domestic and international cases for future planning.

In the data section, the study introduces field survey, traffic flow monitoring, questionnaire, and interview data. The methodology section presents the evaluation framework that integrates operational analysis with sustainability assessment. In the results section, the performance of the loop road in traffic utilization, parcel coordination, and carbon-reduction potential is systematically examined. The discussion section compares Hangzhou’s case with benchmarks such as Wuxi Xidong and Shanghai Zhenru, highlighting gaps and proposing strategies. Finally, the conclusion summarizes the findings and provides practical references for the planning and operation of urban underground transportation systems.

## Methods

### Travel behavior data collection

**Fig 1:**
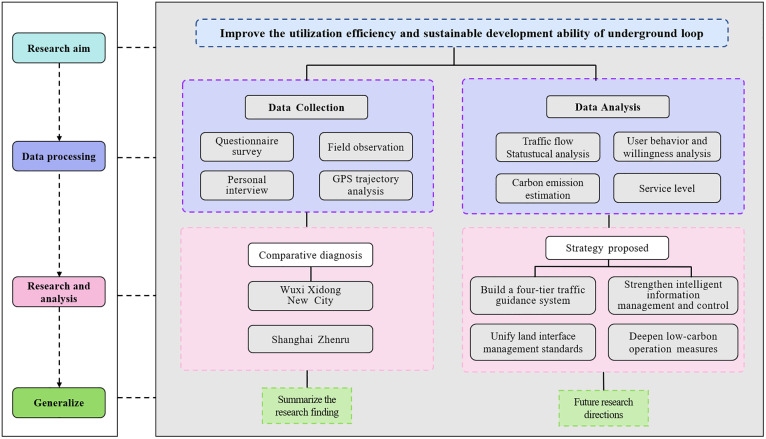
Research framework of the study.

In order to obtain the current usage data of the underground loop of Hangzhou Science and Technology City and analyze its contribution to the sustainable development of the city, this study follows the research framework shown in Fig 1 and uses the following methods to investigate the transit flow, arrival and departure flow of the loop and the willingness of participants to travel.

Questionnaire Survey: The questionnaire survey provided quantitative data on participants’ travel habits and transportation needs, as well as qualitative feedback for optimizing the traffic guidance system and reducing carbon emissions. A total of 360 questionnaires were collected through both online and offline channels, with an effective recovery rate of about 80%. The sample covered various travel groups, including commuters, business travelers, and leisure travelers, ensuring a certain degree of representativeness. All survey participants provided verbal informed consent before answering the questionnaire. The consent process was documented by the interviewer in the survey log and witnessed by a third party.

Observation method: Conduct on-site surveys over five consecutive days across different time periods (covering weekday morning and evening peaks as well as weekends), with observation points set up at nine municipal entrances/exits and fifteen underground garage connection passages. Record directional traffic volumes (pcu/h) and path selection preferences. Combined with surveys of traffic volumes at underground garages within the loop’ s arrival/departure area and at the loop’s municipal entrances/exits, this approach provides data on both arrival-departure and transit traffic volumes.

Individual interviews: In-depth face-to-face interviews with transit and arrival participants to explore their motivations for traveling, the logic of transportation mode choice, and their evaluation of transportation services. The focus was on the participants’ acceptance of the underground loop as a carbon reduction tool and their willingness to increase its use in the future.

GPS Trajectory Analysis: Collect participants’ GPS trajectory data to analyze and simulate their transit and arrival – departure behaviors on and around the loop road. The analysis targets all types of motor vehicles using the underground loop during the survey period (primarily passenger cars), covering weekdays and weekends as well as peak and off-peak periods. Key parameters such as travel time, path selection, average speed, and entrances/exits usage frequency are extracted, and combined with the spatial articulation between the loop road, surface roads, and parcel garage access points to evaluate drivers’ route preferences, travel efficiency, and the loop road’s effectiveness in traffic diversion and carbon reduction.

### Carbon emission estimation

In order to quantitatively evaluate the CO_2_ emissions caused by ground traffic delays and the possible emission reduction effects after the optimization of the underground loop, this study introduces an emission measurement model according to [24]. The total emissions calculation formula is:


$$\begin{array}{c} ef_{FC,CO,{NO}_{x},VOCs,{CH}_{\text{4}},PM}=\frac{\left(\alpha v^{\text{2}}+\beta v+\gamma +\frac{\delta }{v}\right)}{\left(\epsilon v^{\text{2}}+\zeta v+\eta \right)}*(\text{1}-RF) \end{array}$$ (1)

Where efis the pollutant emission factor (in g / (v*km)); α,β,γ,δ,ϵ,ζ,η,v are constants extracted from the *COPERT V* model. These constants vary according to different vehicle types and emission standards; *v* is the speed of the vehicle (in km/h). *RF* is the reduction factor (Reduction Factor), if applicable, for example, in some cases, emissions will be reduced because of some control technology.

The *COPERT V* model is a tool for estimating road traffic pollutant emissions and is widely used in traffic emission research in Europe and China. Its full name is Comprehensive Vehicle Emission Reporting Tool, which is developed by the European Environment Agency and used to calculate the emission factor of pollutants under different road traffic conditions. The model can estimate the emissions of pollutants such as carbon dioxide (CO_2_), nitrogen oxides (NOx), particulate matter (PM), and volatile organic compounds (VOCs) by inputting such as traffic flow, vehicle speed, and road conditions.

The emission factor of CO_2_ is usually calculated based on the emission factor of fuel consumption. The formula is as follows:


$$\begin{array}{c} ef_{CO_{\text{2}}}=\text{3}.\text{1}\text{6}\text{9}*ef_{FC} \end{array}$$ (2)

The CO_2_ emission factor ($ef_{CO_{\text{2}}}$) is obtained by multiplying the emission factor of fuel consumption ($ef_{FC}$). 3.169 is the conversion coefficient between fuel consumption and CO_2_ emissions, reflecting the amount of CO_2_ produced per liter of fuel consumed.

The above formula framework can convert the data obtained from field traffic surveys into carbon emission indicators, thus providing a quantitative basis for assessing the low-carbon contribution of underground loops.

### Ethics statement and field access permissions

Field observations were conducted in publicly accessible transportation areas within the underground loop system of Hangzhou Science and Technology City. No special permits were required for site access or observational data collection.

All participants involved in the questionnaire surveys, interviews, and GPS trajectory data collection voluntarily participated in the study and provided informed verbal consent prior to participation. The consent process was documented by the interviewer in the survey log and witnessed by a third party. No minors were involved in this study. All collected data were anonymized and processed in aggregated form before analysis to protect participants’ privacy.

According to the institutional guidelines of Shanghai Maritime University, this study did not require formal ethical approval because no personally identifiable or clinical information was collected.

## Traffic operation status in the underground loop

**Fig 2:**
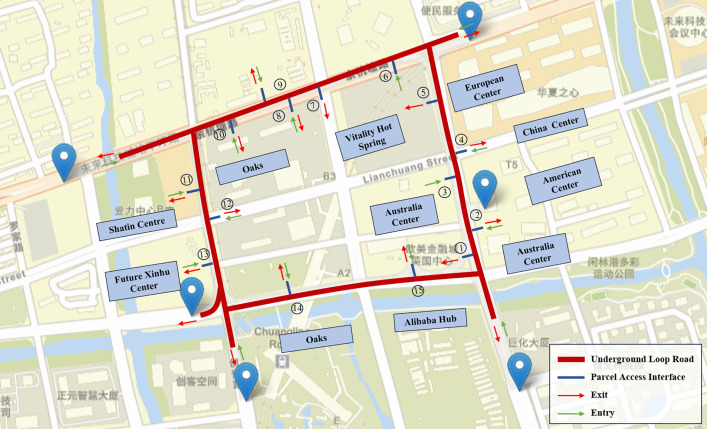
Layout of entrances and exits of the underground loop system.

The underground loop of Hangzhou Future Science and Technology City is located in the core area, with the following geographical scope: east to Liangmu Road, west to Luting Road, south to Wenyi West Road, north to Yuhangtang River, and Metro Line 5 runs through the area. The total area of the planning area is 252.48 hectares, and it is an important municipal infrastructure to serve the transportation from the core area to the development. As shown in Fig 2, the underground loop is about 1.7 km long. Originally, 10 entrances and exits were planned, but the planned entrance on Xiangwang Street East was not constructed. Currently, there are 4 entrances and 5 exits, totaling 9. The loop is directly connected with the expressway and adopts a counterclockwise unidirectional organization mode. Its service object is mainly small cars, taking into account the passage of small firefighting and rescue vehicles, with a design speed of 20 km/h for the main line, 10 km/h for the connection with the underground garage, and a height limit of 3.0m.

### Temporal analysis of traffic operation in the underground loop

#### Analysis of transit and arrival flows in the underground loop.

The survey shows that the current utilization rate of the underground loop in Hangzhou Future Science and Technology City is low. As shown in Table 1, transit traffic accounts for as much as 91% of the total traffic, while arrival and departure traffic only accounts for less than 10%. During peak hours, the transit traffic volume is 824 pcu/h on the surface-level and 949 pcu/h on the underground-level, and vehicles mainly pass through the underground loop directly to the opposite ground level road. During the weekday flat peak hour, weekday traffic volumes decreased by 9 pcu/h compared to weekends, with only a small number of vehicles driving through the loop to underground parking lots in the area to access the various parcels in the planning area.

**Table 1:** Traffic volumes of the underground loop and surrounding region.

Time Period	Area	Transit Traffic Flow (pcu/h)	Arrival/Departure Traffic Flow (pcu/h)
Weekday Peak	Surface-level	824	92
	Underground-level	949	105
Weekday off-Peak	Surface-level	553	46
	Underground-level	602	52
Weekend off-Peak	Surface-level	571	52
	Underground-level	620	55
Percentage of Total Traffic		91.1%	8.9%

Note: “Surface-level” refers to vehicles exiting the loop road to the surface road and “Underground-level” refers to vehicles entering the loop road from the surface road.

The flow distribution data show that the underground loop is currently mainly responsible for the transit channel function, rather than the land parcel arrival and departure service function, and there is a clear functional positioning deviation. This deviation not only leads to the failure to effectively relieve the traffic pressure on the ground but also makes the potential of the loop not fully exploited. A large number of interfaces and parking resources are idle, and there is a mismatch between investment and output. The underlying reasons include insufficient loop connectivity, lack of plot synergy, fault of user information cohesion, and poor reliability of user experience. Subsequent optimization needs to be systematically improved from the aspects of opening up some basement breakpoints, unifying management standards, and building a full-chain information guidance system.

#### Traffic flow analysis of the underground loop connected to parcel access interfaces.

**Fig 3:**
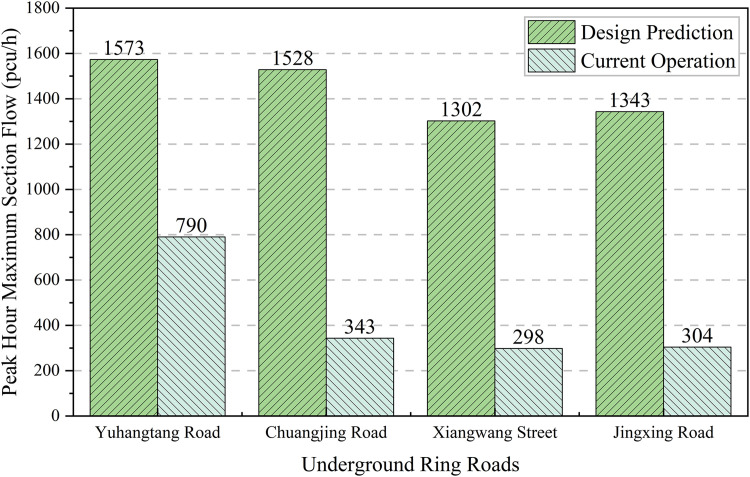
Comparison between designed and observed peak-hour sectional traffic flows.

**Fig 4:**
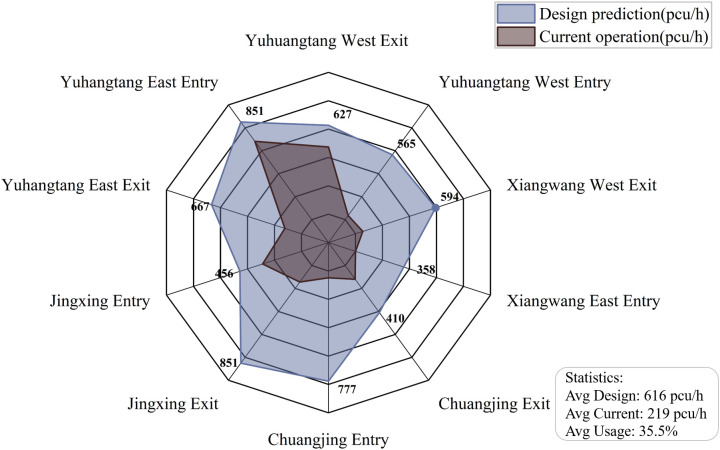
Comparison between designed and observed entrance–exit traffic flows during peak hours.

At present, the flow rate of the underground loop of Hangzhou Future Science and Technology City connecting with the surrounding land is significantly lower than the design expectation, reflecting that its function as a transportation hub from the core area has not yet been effectively activated. Fig 3 shows the maximum cross-section flow of the four sections of the underground loop (Yuhangtang Road, Chuangjing Road, Xiangwang Street, Jingxing Road) during peak hours. The maximum flow rate predicted by the design of Yuhangtang Road is 1573 pcu / h, and the actual operation flow rate is only 790 pcu / h. The other sections are also quite different. Fig 4 shows the flow of the entrance and exit during the peak period. It can be seen that there is a gap between the expected flow and the actual flow of each entrance and exit. Traffic data from the other monitored gate locations show a similar trend, with the overall arrival and departure traffic levels being at a very low level.

In terms of spatial articulation, among the 15 articulated access points, four access points, namely, the garage of the North Building of the Europe-America Plaza – Australia Center, the underground garage of the Future New Lake Center, the underground garage of the South Oaks Center, and the north side of the Yuhangtang Road site are not open at the moment, which further restricts the arrival and departure traffic flow. This indicates that the induction mechanism of the underground loop has the dual challenges of system design flaws and lagging synergistic management of the parcels. In the future, it is necessary to enhance the efficiency and attractiveness of the interface between the underground loop and the parcel access interfaces by optimizing the organization of traffic and enhancing the spatial linkage with the surrounding parcel access interfaces.

#### Analysis of entrance and exit flows between the underground loop and municipal roadways.

Hangzhou Future Science and Technology City underground loop and municipal roads have a total of four pairs of import and export interface, respectively, located in Yuhangtang Road, Xiangwang Street, Chuangjing Road and Jingxing Road. Due to the unidirectional design of the loop road, the inbound traffic is generally greater than the outbound traffic during the morning peak period.

**Fig 5:**
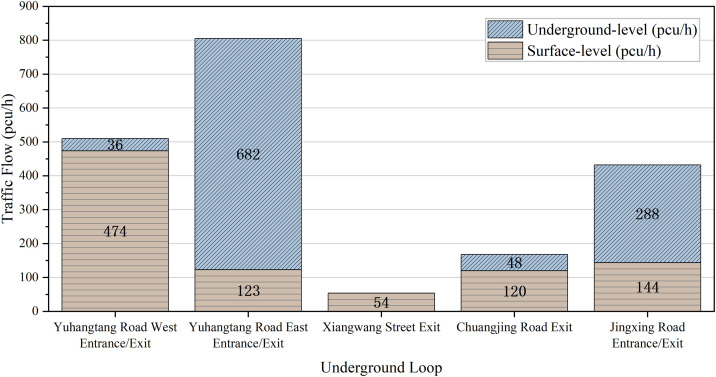
Weekday traffic volumes at entrances and exits of the underground loop and municipal roads.

As shown in Fig 5, the ingress and egress flows are dominated by transit traffic during the peak hour, with relatively small volumes of arriving and departing traffic. During the weekday morning peak hour, the west side entrances/exits of Yuhangtang Road have an upper surface flow of 474 pcu/h and a lower surface flow of 36 pcu/h, a difference of nearly 13 times. Traffic data trends for other entrances and exits are roughly the same, reflecting drivers’ preference to utilize the loop road to quickly traverse the core area rather than to arrive at parcel access interfaces. Transit conditions are more pronounced during the weekday off-peak hours, and the loop’s share of arrival and departure traffic remains low.

Overall, the underground loop has an uneven distribution of entry and exit flows, with a disproportionately high share of transit traffic. This results in the overall operational efficiency of the underground loop not being fully utilized, and at the same time increases the regional carbon emission intensity. In the future, it is necessary to optimize the traffic organization and establish a data sharing mechanism between the road loop operator and the land parcel property to enhance its utilization rate in arrival and departure traffic.

### Statistical analysis of parcel-level traffic flows within the planning area

**Fig 6:**
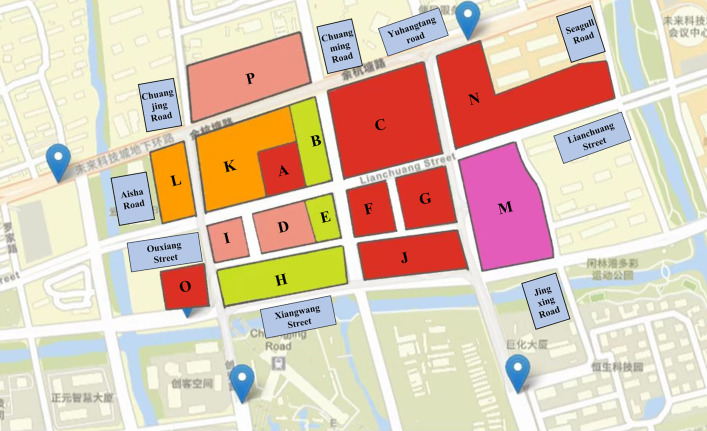
Spatial distribution of parcel access interfaces along the underground loop.

As shown in Fig 6, the loop service plot has multiple functions, mainly covering business (C, G, N, etc.), office (D, M, etc.), hotel apartment (I, L, etc.) and public facilities (B, E, H) and other formats. At present, in addition to the P plot having not yet been built, the remaining plots have been put into use, and the characteristics of traffic flow among different parcels show obvious spatial and temporal differences. Office parcels have prominent tidal traffic phenomenon, with incoming vehicles in the morning peak hour accounting for 75%−85% of the total office traffic throughout the day, while outgoing vehicles in the evening peak hour account for 70%−80%, with highly concentrated commuting demand. The traffic characteristics of commercial complexes are mainly characterized by flexible travel, with a large proportion of temporary visitors, especially on weekends when the flow rises significantly. Among them, holiday traffic in core business districts such as Meitang Plaza surged, with peak traffic increasing by 40%−50% compared to ordinary weekends. Vehicular access flows to and from residential sites are more balanced, but still peak on weekends, highly consistent with residents’ household travel patterns.

The above differences in traffic characteristics reveal the lack of adaptability of the underground loop in serving different types of parcel access interfaces, indicating that the underground transportation system still needs to be improved in meeting diversified travel demands. The next step is to optimize the traffic guidance strategy and operation and management measures of the underground loop in accordance with the specific functions and traffic patterns of the parcel access interfaces, so as to improve the accuracy and effectiveness of the underground loop in serving various types of parcel access interfaces and to better match the diversified traffic demands in the region.

### Service level analysis of the underground loop

**Fig 7:**
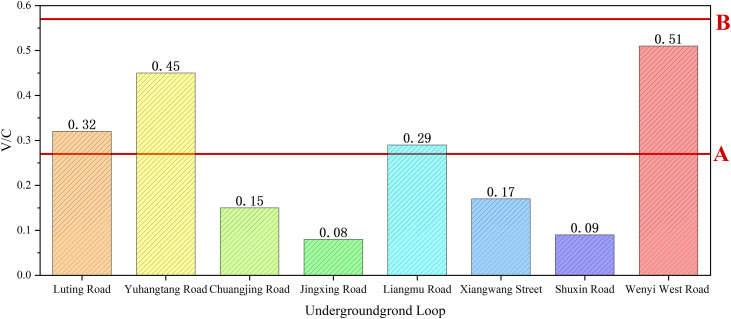
Level-of-service evaluation of municipal roads surrounding the underground loop.

As shown in Fig 7, the overall traffic operation of the underground loop and its surrounding municipal road network in Hangzhou Future Science and Technology City remains relatively efficient. According to the level-of-service (LOS) evaluation criteria for urban signalized intersections based on average vehicle delay (Table 2), major intersections within the core area generally operated at LOS B–C during peak periods, with average vehicle delays ranging from 22.5 s to 34.6 s in the morning peak hour. In addition, based on the road segment service evaluation criteria using the volume-to-capacity ratio (V/C) shown in Table 3, major municipal roads such as Jingxing Road and Shuxin Road generally maintained LOS A or B conditions. During off-peak periods, no significant congestion was observed within the study area.

**Table 2:** Level-of-service criteria for signalized intersections based on average vehicle delay.

Service Level	A	B	C	D	E	F
Average vehicle delay (s)	<10	11 ~ 20	21 ~ 35	36 ~ 55	56 ~ 80	**>**80

**Table 3:** Level-of-service criteria for urban road segments based on volume-to-capacity ratio (V/C).

Service Level	A	B	C	D	E	F
V/C	**≤**0.27	0.27 ~ 0.57	0.57 ~ 0.70	0.70 ~ 0.85	0.85 ~ 1.00	**>**1.0

These findings indicate that the current road planning and traffic operation management strategies are generally capable of meeting daily travel demands while providing relatively smooth travel conditions for drivers and passengers. However, traffic demand is still predominantly concentrated on the surface road system, while the utilization rate of the underground loop remains substantially below the expected level. This phenomenon is mainly attributed to the insufficient effectiveness of the traffic guidance system and information dissemination mechanisms associated with the underground loop. As a result, many drivers have limited awareness and understanding of the underground transportation system, leading to relatively low willingness to use it.

Therefore, future optimization efforts should focus on improving traffic guidance facilities, navigation information delivery, and public awareness of the underground loop system. Enhancing driver familiarity and acceptance of the underground loop could further improve the operational efficiency of the underground transportation system and help alleviate surface-level traffic pressure.

### Analysis of carbon emissions and sustainability impacts of the underground loop

The peak-hour carbon-reduction potential of the underground loop has not yet been fully realized. Existing studies indicate a significant synergistic effect between traffic diversion and carbon reduction [24]. By directing a portion of trips underground, the loop can effectively relieve surface-level congestion and further reduce carbon emissions and noise.

As the core business area (CBD) of Yuhang District, the west-to-east flow on Yuhangtang Road within the Science and Technology City has reached 1,172 pcu/h during peak hours, and congestion with associated delays is expected. At present, the Xiangwang Street-Shuxin Road intersection operates at Service Level B with an average delay of 17.4 s. Using a passenger-car idling CO₂ emission rate of approximately 0.494 g/s, the per-vehicle emission is about 8.6 g/veh.

If the utilization rate of the underground loop line increases, service level improves from B to A, and average delay is reduced to 10.0s, emissions per vehicle will decrease by approximately 3.7 g/veh. Similarly, the Yuhangtang Road-Chuangjing Road intersection currently has a service level of C, with an average delay of 27.3s, corresponding to emissions of about 13.4 g/veh. If the ring road utilization rate increases, service level improves from C to B, and delay time decreases to 20.0s, emissions would fall by about 3.6 g/veh. If service level improves from C to A and delay time decreases to 10.0s, the reduction would be about 8.5 g/veh. These comparisons make clear that the emission reductions mainly arise from shortening surface-level queuing and idling at intersections. Accordingly, the next step is to optimize the traffic guidance system, enhance the loop’s visibility and accessibility, and stabilize peak-hour diversion to further lower surface-level motor-vehicle pollution and support regional green-transportation goals.

## Diagnosis of problems and analysis of countermeasures

To identify the main operational gaps of the Hangzhou Future Science and Technology City underground loop, two representative projects were selected for benchmarking:

Wuxi Xidong New City High-speed Railway Business District: This project has a total length of 2.6 km and adopts a “ring + arc” layout. It contains five entrances and four exits, with graded speed limits of 40 km/h at the ground level and 10 km/h at the articulation points. The design enables rapid distribution of traffic to the high-speed rail hub, and the traffic volume increased fivefold after the project was opened.

Shanghai Zhenru Subcenter H-type Access: This facility is 4 km long and features an H-shaped underground public vehicular access. It includes eight main entrances and exits and connects 12 parcel garages. The “right-in right-out” design is combined with effective left-turn solutions, resulting in a doubling of left-turn traffic flow after opening.

As shown in Table 4, compared with these mature cases, Hangzhou’s underground loop shows lower arrival-departure attraction, weaker guidance, and inconsistent parcel access management.

**Table 4:** Key comparative indicators of three underground access projects.

Aspect	Hangzhou Future Science and Technology City	Wuxi Xidong New City	Shanghai Zhenru Subcenter
Lane Design & Functional Zoning	One-way, 3 lanes (3.0 m width), no clear passing/accel-decel lane division → higher weaving risk	Single lane + distribution lane, graded speed limits, smoother flow	7.5 m two-lane with clear functional zoning, reserved turnaround lane
Connection & Road Network Linkage	Counterclockwise, one-way ramps, some parcel interfaces inconsistent	“Ring + arc” layout, fast linkage to hub & garages	8 main entrances/exits, “right-in right-out” mode reduces surface conflict
Guidance System & Info Transfer	LED inside loop, no preview signs at key intersections, navigation recommendation <10%	Clear layout with entrance preview signs	Standardized signage linked with surface traffic lights, high driver awareness
Operational Synergy & Resource Integration	4 of 15 parcel connections unopened, inconsistent management	Fully integrated core area garages, unified operation	12 parcel garages connected, unified operation, high utilization

## Discrepancy between user awareness and actual experience

**Fig 8:**
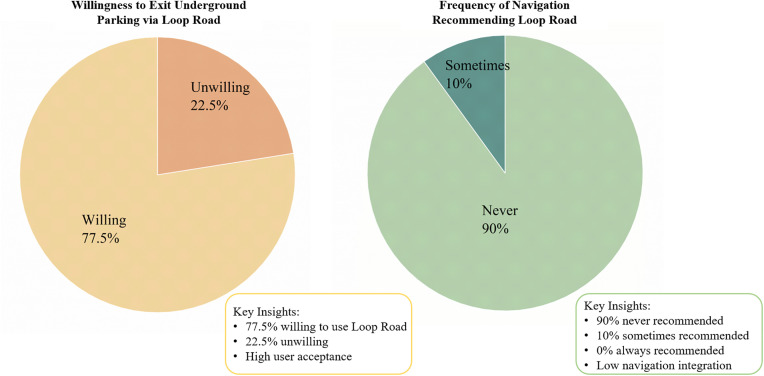
Survey results on underground loop usage behavior and navigation preferences.

The recommendation rate of the underground loop of Hangzhou Future Science and Technology City in the navigation software is less than 10%, and the underground GPS signal is weak. The lack of advance notice boards at peripheral intersections leads some drivers to give up their choices due to incomplete information even if they intend to use them. According to the results of the questionnaire survey, 77.5% of respondents are willing to leave the underground parking lot through the underground loop, but the navigation software recommends the underground loop less frequently. 90% of respondents almost never received the recommendation, and only 10% said they received it occasionally (Fig 8).

Drawing on these experiences, the loop road should leverage intelligent traffic management to disseminate real-time traffic conditions and available parking spaces through multiple channels, and install tiered directional signage at peripheral intersections, major feeder roads, and key nodes inside and outside the loop. At the same time, stable data interfaces should be established with navigation platforms such as Gaode and Baidu to enable real-time pushing of loop conditions and parking information, ensuring that drivers receive accurate and timely information both before and during their trip, thereby increasing utilization and improving operational efficiency.

## Limitations in the traffic guidance system and information accuracy

At present, there are obvious deficiencies in the external guidance of Hangzhou underground loop: the surrounding intersections and peripheral municipal roads lack advance notice, the guidance facilities are mostly concentrated at the entrance, the internal channel does not set clear guidance signs, and the entrance and exit information is also more general. The peak hour actual flow (790 pcu/h) of the Yuhangtang section is only 50.2% of the design expectation (1573 pcu/h), and there is also a huge gap between other sections and entrances and exits. This directly leads to the fact that the loop mainly undertakes the passing function, and its transit traffic accounts for as high as 91.1%, while the arrival and departure service function fails to play effectively.

To address this gap, a four-tier traffic guidance system can be developed. Level 1 guidance is deployed at upstream intersections of loop entrances and exits (e.g., the intersection of Yuhangtang Road and Luting Road), using variable message signs to display real-time traffic conditions on main roads and the loop, guiding vehicles to plan entry routes in advance. For example, during the morning peak period, the optimal entrance route from Wenyi West Road to the underground loop can be displayed in real time to reduce surface-road detours.

Level 2 guidance adopts a “single lane and distribution lane” cross-section, covering connecting roads such as Chuangjing Road and Jingxing Road, and releases information on loop traffic density and parking-lot locations. Drawing on the experience of Wuxi Xidong New City, LED screens can be installed before ramp entrances to provide rolling prompts such as “Yuhangtang Road entrance congested, consider entering via Jingxing Road.”

Level 3 guidance is set at parcel garage entrances such as Europe-America Plaza and Oaks Center, clearly marking the parcel name and remaining parking spaces—for example, at Meitang Plaza, displaying “Loop directly to B3, 120 spaces remaining”.

Level 4 guidance is embedded inside garages, linking parking guidance systems with loop-exit information to optimize vehicle departure routes—for example, inside the Australian Center garage, displaying “Exit via Xiangwang Street, approx. 3 minutes”.

This progressive layout, moving from distant to near and step by step, ensures that participants receive clear and dynamically updated information before entering, while traveling within, and when exiting the loop. It can significantly increase the proportion of arrival-departure traffic and enhance the loop’s role in diverting surface traffic.

## Low efficiency in parcel coordination and resource utilization

The dual short board of physical connection and management coordination is the core bottleneck restricting the traffic function of the loop. Four of the 15 connecting channels of the underground loop of Hangzhou Science and Technology City are not open yet, and the open interfaces lack uniformity in management standards. The occupancy rate of some plots in the core area needs to be improved. The occupancy rate of the Oaks Center (D and I plots) is 30%−35%, and the occupancy rate of the New Lake Center (O plot) is only 30% in the future. In contrast, Wuxi and Shanghai have achieved interface standardization and land garage interconnection, enabling vehicles to flow efficiently between plots and significantly improving resource utilization.

To address this, the occupancy rate of parcels in the planning area should be increased, introducing supporting services such as vehicle maintenance, car washing, and charging stations(2). Interface management standards should be unified, physical interconnection between garages promoted, and unopened connections gradually made operational. In addition, a joint scheduling mechanism between loop entrances/exits and parcel access interfaces should be established to improve parking-space sharing, enhance vehicle circulation, and increase both attractiveness and efficiency.

## Inadequate sustainability and carbon reduction outcomes

At present, the utilization rate of the underground loop is not as expected, the loop diversion effect is limited, and the carbon reduction benefit has not been realized on a large scale. If the service level of Xiangyuan Street-Shuxin Road intersection is raised from B to A, 3.7 g CO_2_ emission per vehicle can be reduced. The average transit traffic volume (about 886.5 pcu/h) during the peak hour of the working day is taken, and the average delay is assumed to be 25s. The CO_2_ emissions per vehicle are 12.35 g/veh, and the additional CO_2_ emissions of the ground road network in the core area due to vehicle idling during peak hours are 10950 g/h. At the same time, the continuous operation of lighting, ventilation and other systems increases energy consumption and fails to give full play to the intensive operation advantages of underground systems. International advanced cases generally combine dynamic carbon emission monitoring with green energy utilization to enhance sustainable operation capabilities.

To improve outcomes, a dynamic carbon-emission monitoring and management mechanism should be established, linking usage frequency directly to emission-reduction effects and visualizing data in real time. Solar panels should be installed on entrance canopies to power lighting, and LED intelligent sensor lights should replace existing fixtures. Parking-discount policies can be applied for drivers who transfer to public transport after using the loop, encouraging green travel and achieving both energy saving and carbon reduction.

## Limited operational flexibility and emergency response capability

The underground loop in Hangzhou adopts a one-way traffic organization mode. Although this design reduces traffic conflicts, it increases the average detour distance by about 1.5–2.0 km. GPS trajectory analysis shows that during off-peak hours, about 35% of drivers who are familiar with the road conditions choose the ground road, resulting in idle loop resources. In contrast, Wuxi and Shanghai pay more attention to the traffic efficiency of different vehicle types in traffic organization design, and flexibly adjust the traffic organization mode when necessary.

To improve, the connection routes between the loop and parcels should be optimized to reduce detours while retaining the advantages of one-way flow. Real-time monitoring of traffic conditions on the loop and connecting surface roads should be implemented, with signal coordination and event-response mechanisms established. These measures would enable flow restrictions and targeted guidance during incidents, reducing both the scope and severity of impacts on surrounding roads.

## Conclusion

This study investigated the current operational performance of the underground loop system in Hangzhou Future Science and Technology City and identified the dual challenges of low utilization efficiency and insufficient realization of sustainability benefits. Comparative analyses with the underground vehicular access systems in the high-speed railway business district of Xidong New City in Wuxi and the H-type underground vehicular system in the Zhenru Subcenter in Shanghai further demonstrated the influence of lane configuration and connection forms on traffic efficiency and environmental performance. The results indicate that the low utilization rate of the underground loop is mainly associated with the low occupancy rate of parcel access interfaces, inconsistent interface management standards, and insufficient effectiveness of the traffic guidance system.

To address these issues, this study proposes several optimization strategies, including the establishment of a comprehensive four-level traffic guidance system, enhancement of intelligent traffic information dissemination and control, coordinated management of parcel access interfaces, and promotion of low-carbon operational management. These measures could improve the operational efficiency and sustainability performance of underground loop systems, while also alleviating surface traffic pressure, enhancing regional transportation efficiency, and supporting the sustainable development of urban core areas.
